# Supplementary material for: Psychological safety and patient safety: A systematic and narrative review
Source: PLoS One. 2025 Apr 24;20(4):e0322215. doi: 10.1371/journal.pone.0322215 (PMC12021220; doi:10.1371/journal.pone.0322215)
Supplement: S2 File — (DOCX) [file pone.0322215.s002.docx]

Supplemental material 2

Search strategy

**Manuscript: Psychological Safety and Patient Safety: a systematic and narrative review**

**Supplementary Table 1. Search Strings for multiple databases**

1. *Search strategy*

*Exposure variable:* a. (“psychological safety*” OR “team safety” OR “team safety climate”)

**AND**

*Outcome variable:* b1. (“patient safety” OR “patient safety climate” OR “patient safety culture” OR “quality of care”)

b2. (“patient mortality” OR “medical errors” OR “safety concern*” OR “patient morbidity*” OR “safety climate*” OR “near-misses”)

b3. (“AHRQ” OR “HSOPSC” OR “HSPSC” OR “MaPSaF” OR “MOSPSC” OR “MSI-2006” OR “PSCHO” OR “PCISME” OR “SAQ” OR “SOS” OR “SHSQ” OR “VSCQ” OR)

b4. (“Agency for Healthcare Research and Quality” OR “Hospital Survey on Patient Safety Culture” OR “Manchester Patient Safety Framework” OR “Medical Office Survey on Patient Safety Culture” OR “Modified Stanford Instrument” OR “Patient Safety Climate in Health Care Organizations” OR “Primary Care International Study of Medical Errors” OR “Safety Attitudes Questionnaire” OR “Safety Organizing Scale” OR “Scottish Hospital Safety Questionnaire” OR “Vienna Safety Climate Questionnaire”)

*Supplementary Table 1. Search Strings for multiple databases*

| *Database* | *Query* |  | *Results* |
| --- | --- | --- | --- |
| Web of Science | (AB=(“psychological safety*” OR “team safety” OR “team safety climate”)) |  |  |
|  | **AND** |  |  |
|  | (AB=(“patient safety” OR “patient safety climate” OR “patient safety culture” OR “quality of care” OR “patient mortality” OR “medical errors” OR “safety concern*” OR “patient morbidity*” OR “safety climate*” OR “near-misses” OR “AHRQ” OR “HSOPSC” OR “HSPSC” OR “MaPSaF” OR “MOSPSC” OR “MSI-2006” OR “PSCHO” OR “PCISME” OR “SAQ” OR “SOS” OR “SHSQ” OR “VSCQ” OR “Agency for Healthcare Research and Quality” OR “Hospital Survey on Patient Safety Culture” OR “Manchester Patient Safety Framework” OR “Medical Office Survey on Patient Safety Culture” OR “Modified Stanford Instrument” OR “Patient Safety Climate in Health Care Organizations” OR “Primary Care  International Study of Medical Errors” OR “Safety Attitudes Questionnaire” OR “Safety Organizing Scale” OR “Scottish Hospital Safety Questionnaire” OR “Vienna Safety Climate Questionnaire”)) |  |  |
| PubMed | (“psychological safety”[Title/Abstract] OR “team safety”[Title/Abstract] OR “team safety climate”[Title/Abstract]) |  |  |
|  | **AND** |  |  |
|  | (“patient safety”[Title/Abstract] OR “patient safety climate”[Title/Abstract] OR “patient safety culture”[Title/Abstract] OR “quality of care”[Title/Abstract] OR “patient mortality”[Title/Abstract] OR “medical errors”[Title/Abstract] OR “safety concern*”[Title/Abstract] OR “patient morbidity*”[Title/Abstract] OR “safety climate*”[Title/Abstract] OR “near-misses”[Title/Abstract]) OR “AHRQ”[Title/Abstract] OR “HSOPSC”[Title/Abstract] OR “HSPSC”[Title/Abstract] OR “MaPSaF”[Title/Abstract] OR “MOSPSC”[Title/Abstract] OR “MSI-2006”[Title/Abstract] OR “PSCHO” [Title/Abstract] OR “PCISME” [Title/Abstract] OR “SAQ” [Title/Abstract] OR “SOS”[Title/Abstract] OR “SHSQ”[Title/Abstract] OR “VSCQ”[Title/Abstract] OR “Agency for Healthcare Research and Quality”[Title/Abstract] OR “Hospital Survey on Patient Safety Culture”[Title/Abstract] OR “Manchester Patient Safety Framework”[Title/Abstract] OR “Medical Office Survey on Patient Safety Culture” [Title/Abstract] OR “Modified Stanford Instrument”[Title/Abstract] OR “Patient Safety Climate in Health Care Organizations”[Title/Abstract] OR “Primary Care  International Study of Medical Errors”[Title/Abstract] OR “Safety Attitudes Questionnaire” OR “Safety Organizing Scale”[Title/Abstract] OR “Scottish Hospital Safety Questionnaire”[Title/Abstract] OR “Vienna Safety Climate Questionnaire”[Title/Abstract]) |  |  |
| PsycINFO via OVID  (Limits abstract; peer review; article) | (("psychological safety*" or "team safety" or "team safety climate") |  |  |
|  | **AND** |  |  |
|  | ("patient safety" or "patient safety climate" or "patient safety culture" or "quality of care" or "patient mortality" or "medical errors" or "safety concern" or "patient morbidity" or "near-misses" or ahrq or hsopsc or hspsc or mapsaf or mospsc or "MSI-2006" or pscho or pcisme or saq or sos or shsq or vscq or "Agency for Healthcare Research and Quality" or "Hospital Survey on Patient Safety Culture" or "Manchester Patient Safety Framework" or "Medical Office Survey on Patient Safety Culture" or "Modified Stanford Instrument" or "Patient Safety Climate in Health Care Organizations" or "Primary Care International Study of Medical Errors" or "Safety Attitudes Questionnaire" or "Safety Organizing Scale" or "Scottish Hospital Safety Questionnaire" or "Vienna Safety Climate Questionnaire")).ab. |  |  |
| Scopus | TITLE-ABS-KEY (“psychological safety*” OR “team safety” OR “team safety climate”) |  |  |
|  | **AND** |  |  |
|  | TITLE-ABS-KEY (“patient safety” OR “patient safety climate” OR “patient safety culture” OR “quality of care” OR “patient mortality” OR “medical errors” OR “safety concern*” OR “patient morbidity*” OR “safety climate*” OR “near-misses” OR “AHRQ” OR “HSOPSC” OR “HSPSC” OR “MaPSaF” OR “MOSPSC” OR “MSI-2006” OR “PSCHO” OR “PCISME” OR “SAQ” OR “SOS” OR “SHSQ” OR “VSCQ” OR “Agency for Healthcare Research and Quality” OR “Hospital Survey on Patient Safety Culture” OR “Manchester Patient Safety Framework” OR “Medical Office Survey on Patient Safety Culture” OR “Modified Stanford Instrument” OR “Patient Safety Climate in Health Care Organizations” OR “Primary Care International Study of Medical Errors” OR “Safety Attitudes Questionnaire” OR “Safety Organizing Scale” OR “Scottish Hospital Safety Questionnaire” OR “Vienna Safety Climate Questionnaire”) |  |  |
| Embase via OVID  (Limits abstract; peer review; article) | (("psychological safety*" or "team safety" or "team safety climate") |  |  |
|  | **AND** |  |  |
|  | ("patient safety" or "patient safety climate" or "patient safety culture" or "quality of care" or "patient mortality" or "medical errors" or "safety concern" or "patient morbidity" or "near-misses" or ahrq or hsopsc or hspsc or mapsaf or mospsc or "MSI-2006" or pscho or pcisme or saq or sos or shsq or vscq or "Agency for Healthcare Research and Quality" or "Hospital Survey on Patient Safety Culture" or "Manchester Patient Safety Framework" or "Medical Office Survey on Patient Safety Culture" or "Modified Stanford Instrument" or "Patient Safety Climate in Health Care Organizations" or "Primary Care International Study of Medical Errors" or "Safety Attitudes Questionnaire" or "Safety Organizing Scale" or "Scottish Hospital Safety Questionnaire" or "Vienna Safety Climate Questionnaire")).ab. |  |  |
| Cochrane Library  (Limits title& abstract; peer review; article) | (“psychological safety*” OR “team safety” OR “team safety climate”) |  |  |
|  | **AND** |  |  |
|  | (“patient safety” OR “patient safety climate” OR “patient safety culture” OR “quality of care” OR “patient mortality” OR “medical errors” OR “safety concern*” OR “patient morbidity*” OR “safety climate*” OR “near-misses” OR “AHRQ” OR “HSOPSC” OR “HSPSC” OR “MaPSaF” OR “MOSPSC” OR “MSI-2006” OR “PSCHO” OR “PCISME” OR “SAQ” OR “SOS” OR “SHSQ” OR “VSCQ” OR “Agency for Healthcare Research and Quality” OR “Hospital Survey on Patient Safety Culture” OR “Manchester Patient Safety Framework” OR “Medical Office Survey on Patient Safety Culture” OR “Modified Stanford Instrument” OR “Patient Safety Climate in Health Care Organizations” OR “Primary Care International Study of Medical Errors” OR “Safety Attitudes Questionnaire” OR “Safety Organizing Scale” OR “Scottish Hospital Safety Questionnaire” OR “Vienna Safety Climate Questionnaire”) |  |  |
| CINAHL via EBSCOhost | (“psychological safety*” OR “team safety” OR “team safety climate”) |  |  |
|  | **AND** |  |  |
|  | (“patient safety” OR “patient safety climate” OR “patient safety culture” OR “quality of care” OR “patient mortality” OR “medical errors” OR “safety concern*” OR “patient morbidity*” OR “safety climate*” OR “near-misses” OR “AHRQ” OR “HSOPSC” OR “HSPSC” OR “MaPSaF” OR “MOSPSC” OR “MSI-2006” OR “PSCHO” OR “PCISME” OR “SAQ” OR “SOS” OR “SHSQ” OR “VSCQ” OR “Agency for Healthcare Research and Quality” OR “Hospital Survey on Patient Safety Culture” OR “Manchester Patient Safety Framework” OR “Medical Office Survey on Patient Safety Culture” OR “Modified Stanford Instrument” OR “Patient Safety Climate in Health Care Organizations” OR “Primary Care International Study of Medical Errors” OR “Safety Attitudes Questionnaire” OR “Safety Organizing Scale” OR “Scottish Hospital Safety Questionnaire” OR “Vienna Safety Climate Questionnaire”) |  |  |
| Google Scholar (Limits: Title) | (“psychological safety*” OR “team safety” OR “team safety climate”) |  |  |
|  | **AND** |  |  |
|  | (“patient safety” OR “patient safety climate” OR “patient safety culture”) |  |  |
|  | (“patient mortality” OR “medical errors” OR “patient morbidity*” OR “near-misses”) |  |  |
|  | (“quality of care”) |  |  |
|  | (“AHRQ” OR “HSOPSC” OR “HSPSC” OR “MaPSaF”) |  |  |
|  | (“MOSPSC” OR “MSI-2006” OR “PSCHO” OR “PCISME”) |  |  |
|  | (“SAQ” OR “SOS” OR “SHSQ” OR “VSCQ”) |  |  |
|  | (“Agency for Healthcare Research and Quality” OR “Hospital Survey on Patient Safety Culture” OR “Manchester Patient Safety Framework”) |  |  |
|  | (“Medical Office Survey on Patient Safety Culture” OR “Modified Stanford Instrument” OR “Patient Safety Climate in Health Care Organizations”) |  |  |
|  | (“Primary Care International Study of Medical Errors” OR “Safety Attitudes Questionnaire” OR “Safety Organizing Scale”) |  |  |
|  | “Scottish Hospital Safety Questionnaire” OR “Vienna Safety Climate Questionnaire”) |  |  |
