# Supplementary material for: Psychological safety and patient safety: A systematic and narrative review
Source: PLoS One. 2025 Apr 24;20(4):e0322215. doi: 10.1371/journal.pone.0322215 (PMC12021220; doi:10.1371/journal.pone.0322215)
Supplement: S3 File — (DOCX) [file pone.0322215.s003.docx]

*Supplementary Material 3. Inclusion and exclusion criteria*

|  | Inclusion criteria | Exclusion criteria |
| --- | --- | --- |
| Population | Physicians/Doctors, including Residents, Clinicians, Nurses, Head nurses, Nurses assistants, Registered Nurses/Licensed Nurses, charge Nurses Non-physician and Non-clinicians healthcare professionals/providers working in patient-care units/hospitals (e.g., paramedics, respiratory therapists, patient-care technicians, unit secretaries, managers, assistant managers, educators, coordinators or administrative staff/ workers, technicians/medical laboratory scientists, data analysts, supervisors or managers, medical assistants, social workers, directors, pharmacists/pharmaceutics, clinical psychologists, physiotherapists, ambulance technicians, medical assistants) Articles about a wide range of healthcare workers only if they work patient-care units, impatient settings, mental health units/hospitals, hospital system including teaching and non-teaching hospitals from various divisions and specialty departments to ensure that the sample is representative of the population studied to avoid selection bias. | Articles about medical students.  Articles including multiple professional groups and do not provide specific data on health and social care workers. |
| Type of publication | Studies with quantitative methodology, cross sectional studies with and without control groups, longitudinal methodology with and without control groups, mixed methodology with and without control groups, interventions | Narrative reviews, editorials, letters to the editor, conference abstracts/papers, studies with qualitative methodology, scoping reviews and systematic reviews/meta-analysis, randomized clinical trials, thesis/dissertation |
| Condition or domain | Full-text articles.  Only studies providing specific data about the relationship between psychological safety and patient safety outcomes using robust and objective measures of safety with validity support from commonly accepted sources of evidence. | Studies estimating or considering patient safety based on subjective assessments/self-report data (experience perceptions and willingness relies on individual judgments and decisions leading to common report bias or social desirability bias).  Studies that do not use reliable and valid measures.  Studies that did not measure at least one of the desired outcomes. |
| Interventions | Any interventions targeting healthcare providers or health services more broadly focused on clinician/non-clinician behaviors or skills, team processes, professional support, education or training, organizational change processes, or any other interventions that are related to patient safety outcomes. | None |
| Language | Only English | Other languages |
| Date | no time restriction | None |
