# Supplementary material for: Psychological safety and patient safety: A systematic and narrative review
Source: PLoS One. 2025 Apr 24;20(4):e0322215. doi: 10.1371/journal.pone.0322215 (PMC12021220; doi:10.1371/journal.pone.0322215)
Supplement: S4 File — (PDF) [file pone.0322215.s004.pdf]

Study	***Reasons for inclusion and exclusion***
1. Adair, K. C., Heath, A., Frye, M. A., Frankel, A., Proulx, J., Rehder, K. J., ... & Sexton, J. B. (2022). The Psychological Safety Scale of the Safety, Communication, Operational, Reliability, and Engagement (SCORE) Survey: a brief, diagnostic, and actionable metric for the ability to speak up in healthcare settings. *Journal of patient safety*, *18*(6), 513-520.	Self-report*
2. Adair, K. C., Levoy, E., Tawfik, D. S., Palassof, S., Profit, J., Frankel, A., ... & Sexton, J. B. (2023). Assessing leadership behavior in health care: Introducing the local leadership scale of the SCORE survey. *The Joint Commission Journal on Quality and Patient Safety*, *49*(3), 166-173.	No measure of PS
3. Alingh, C. W., Van Wijngaarden, J. D. H., Van De Voorde, K., Paauwe, J., & Huijsman, R. Speaking up about patient safety concerns: the influence of safety management approaches and climate on nurses' willingness to speak up. BMJ Qual Saf. 2019; 28 (1): 39–48.	Self-report*
4. Anderson, E., Mohr, D. C., Regenbogen, I., Swamy, L., Smith, E. G., Mourra, S., & Rinne, S. T. (2021). Influence of organizational climate and clinician morale on seclusion and physical restraint use in inpatient psychiatric units. Journal of Patient Safety, 17(4), 316-322.	Objective measure of neglect
5. Appelbaum, N. P., Dow, A., Mazmanian, P. E., Jundt, D. K., & Appelbaum, E. N. (2016). The effects of power, leadership and psychological safety on resident event reporting. *Medical education*, *50*(3), 343-350.	Self-report PS
6. Arad, D., & Finkelstein, A. (2022). Patient safety and staff psychological safety: a mixed methods study on aspects of teamwork in the operating room. *Frontiers in public health*, *10*, 1060473.	Wrong outcome
7. Arnetz, J., Sudan, S., Goetz, C., Counts, S., & Arnetz, B. (2019). Nurse work environment and stress biomarkers: Possible implications for patient outcomes. Journal of occupational and environmental medicine, 61(8), 676-681.	Objective measure of PS
8. Bradley, E. H., Brewster, A. L., Fosburgh, H., Cherlin, E. J., & Curry, L. A. (2017). Development and psychometric properties of a scale to measure hospital organizational culture for cardiovascular	Not enough info on PS and QoC

care. *Circulation: Cardiovascular Quality and Outcomes*, *10*(3), e003422.	
9. Brborović, O., Brborović, H., & Hrain, L. (2022). The COVID-19 pandemic crisis and patient safety culture: a mixed-method study. *International journal of environmental research and public health*, *19*(4), 2237.	No measure of Psych Safety
10. Brimhall, K. C., Tsai, C. Y., Eckardt, R., Dionne, S., Yang, B., & Sharp, A. (2023). The effects of leadership for self-worth, inclusion, trust, and psychological safety on medical error reporting. Health Care Management Review, 48(2), 120-129.	Objective measure of PS
11. Bronkhorst, B. (2015). Behaving safely under pressure: The effects of job demands, resources, and safety climate on employee physical and psychosocial safety behavior. *Journal of safety research*, *55*, 63-72.	Self-report*
12. Buljac-Samardžić, M., Dekker-van Doorn, C., & Van Wijngaarden, J. (2021). Detach yourself: The positive effect of psychological detachment on patient safety in long-term care. *Journal of patient safety*, *17*(7), 490-496.	Self-report*
13. Cartland, J., Green, M., Kamm, D., Halfer, D., Brisk, M. A., & Wheeler, D. (2022). Measuring psychological safety and local learning to enable high reliability organisational change. *BMJ Open Quality*, *11*(4), e001757.	No measure of PS
14. Cho, H., Steege, L. M., & Arsenault Knudsen, É. N. (2023). Psychological safety, communication openness, nurse job outcomes, and patient safety in hospital nurses. *Research in Nursing & Health*, *46*(4), 445-453.	Self-report*
15. Clark, O. L., Zickar, M. J., & Jex, S. M. (2014). Role definition as a moderator of the relationship between safety climate and organizational citizenship behavior among hospital nurses. *Journal of Business and Psychology*, *29*, 101-110.	No measure of PS
16. Colet, P. C., Cruz, J. P., Cacho, G., Al-Qubeilat, H., Soriano, S. S., & Cruz, C. P. (2018). Perceived infection prevention climate and its predictors among nurses in Saudi Arabia. *Journal of Nursing Scholarship*, *50*(2), 134-142.	Self-report*
17. Derickson, R., Fishman, J., Osatuke, K., Teclaw, R., & Ramsel, D. (2015). Psychological safety and error reporting within Veterans Health Administration hospitals. *Journal of patient safety*, *11*(1), 60-66.	Self-report*

18. Dieckmann, P., Tulloch, S., Dalgaard, A. E., & Varming, K. (2022). Psychological safety during the test of new work processes in an emergency department. *BMC Health Services Research*, *22*(1), 307.	No Patient Safety outcome
19. Dietl, J. E., Derksen, C., Keller, F. M., & Lippke, S. (2023). Interdisciplinary and interprofessional communication intervention: How psychological safety fosters communication and increases patient safety. *Frontiers in Psychology*, *14*, 1164288.	Self-report*
20. Dietl, J. E., Derksen, C., Keller, F. M., Schmiedhofer, M., & Lippke, S. (2023). Psychosocial Processes in Healthcare Workers: How Individuals' Perceptions of Interpersonal Communication Is Related to Patient Safety Threats and Higher-Quality Care. *International Journal of Environmental Research and Public Health*, *20*(9), 5698.	Self-report*
21. Eldor, L., Hodor, M., & Cappelli, P. (2023). The limits of psychological safety: Nonlinear relationships with performance. *Organizational Behavior and Human Decision Processes*, *177*, 104255.	No measure of PS
22. Erkutlu, H., & Chafra, J. (2019). Leader psychopathy and organizational deviance: the mediating role of psychological safety and the moderating role of moral disengagement. *International Journal of Workplace Health Management*, *12*(4), 197-213.	No measure of PS
23. Etezad, S., Fleming, M., Weigand, H. A., Hartt, C. M., Dutton, D. J., Barker, J. R., & Brunt, K. R. (2023). Exploring the well-being of community pharmacy professionals, turnover intention and patient safety: Time to include operational responsibility. *Canadian Pharmacists Journal/Revue des Pharmaciens du Canada*, *156*(2), 71-84.	Self-report*
24. Falcone, M. L., Tokac, U., Fish, A. F., Van Stee, S. K., & Werner, K. B. (2023). Factor Structure and Construct Validity of a Hospital Survey on Patient Safety Culture Using Exploratory Factor Analysis. *Journal of Patient Safety*, *19*(5), 323-330.	self-report
25. Gazica, M. W., & Spector, P. E. (2016). A test of safety, violence prevention, and civility climate domain-specific relationships with relevant workplace hazards. *International journal of occupational and environmental health*, *22*(1), 45-51.	No Patient Safety outcome
26. Giallonardo, L. M. (2020). *Unit Managers' Authentic Leadership, Staff Nurses' Work Attitudes and Behaviours, and Outcomes of Care: A Structural*	Self-report*

Equation Model (Doctoral dissertation, The University of Western Ontario (Canada)).	
27. Gilmartin, H. M., Langner, P., Gokhale, M., Osatuke, K., Hasselbeck, R., Maddox, T. M., & Battaglia, C. (2018). Relationship between psychological safety and reporting nonadherence to a safety checklist. Journal of nursing care quality, 33(1), 53-60.	Objective measure of PS
28. Gilmartin, H. M., Saint, S., Ratz, D., Chrouser, K., Fowler, K. E., & Greene, M. T. (2023). The influence of hospital leadership support on burnout, psychological safety, and safety climate for US infection preventionists during the coronavirus disease 2019 (COVID-19) pandemic. *Infection Control & Hospital Epidemiology*, 1-6.	Self-report
29. Greene, M. T., Gilmartin, H. M., & Saint, S. (2020). Psychological safety and infection prevention practices: Results from a national survey. *American journal of infection control*, *48*(1), 2-6.	Self-report*
30. Habibi Soola, A., Ajri-Khameslou, M., Mirzaei, A., & Bahari, Z. (2022). Predictors of patient safety competency among emergency nurses in Iran: a cross-sectional correlational study. *BMC health services research*, *22*(1), 547.	Self-report PS*
31. Halbesleben, J. R., & Rathert, C. (2008). The role of continuous quality improvement and psychological safety in predicting work-arounds. *Health care management review*, *33*(2), 134-144.	Self-report*
32. Halbesleben, J. R., Leroy, H., Dierynck, B., Simons, T., Savage, G. T., McCaughey, D., & Leon, M. R. (2013). Living up to safety values in health care: The effect of leader behavioral integrity on occupational safety. Journal of Occupational Health Psychology, 18(4), 395.	Objective measure of PS
33. Han, J. H., & Roh, Y. S. (2020). Teamwork, psychological safety, and patient safety competency among emergency nurses. *International Emergency Nursing*, *51*, 100892.	Self-report*
34. Harrison, R., Manias, E., Ellis, L., Mimmo, L., Walpola, R., Roxas-Harris, B., ... & Hay, L. (2022). Evaluating clinician experience in value-based health care: the development and validation of the Clinician Experience Measure (CEM). *BMC Health Services Research*, *22*(1), 1484.	self report QoC
35. Hirak, R., Peng, A. C., Carmeli, A., & Schaubroeck, J. M. (2012). Linking leader inclusiveness to work unit performance: The importance of psychological	Self-report*

safety and learning from failures. *The Leadership Quarterly*, *23*(1), 107-117.	
36. Hu, X., & Casey, T. (2021). How and when organization identification promotes safety voice among healthcare professionals. *Journal of Advanced Nursing*, *77*(9), 3733-3744.	Self-report
37. Jimmieson, N. L., Tucker, M. K., White, K. M., Liao, J., Campbell, M., Brain, D., ... & Graves, N. (2016). The role of time pressure and different psychological safety climate referents in the prediction of nurses' hand hygiene compliance. *Safety Science*, *82*, 29-43.	self-report
38. Jung, O. S., Kundu, P., Edmondson, A. C., Hegde, J., Agazaryan, N., Steinberg, M., & Raldow, A. (2021). Resilience vs. vulnerability: psychological safety and reporting of near misses with varying proximity to harm in radiation oncology. The Joint Commission Journal on Quality and Patient Safety, 47(1), 15-22.	Used Vignettes to measure patients safety
39. Lavelle, M., Darzi, A., Starodub, R., & Anderson, J. E. (2022). The role of transactive memory systems, psychological safety and interpersonal conflict in hospital team performance. *Ergonomics*, *65*(3), 519-529.	Self-report*
40. Lee, S. E., & Dahinten, V. S. (2021). Psychological safety as a mediator of the relationship between inclusive leadership and nurse voice behaviors and error reporting. *Journal of Nursing Scholarship*, *53*(6), 737-745.	Self-report*
41. Lee, S. E., Dahinten, V. S., & Lee, J. H. (2023). Testing the association between the enabling and enacting factors of patient safety culture and patient safety: structural equation modelling. *BMC nursing*, *22*(1), 32.	Self-report*
42. Lee, Y. H., Yang, C. C., & Chen, T. T. (2016). Barriers to incident-reporting behavior among nursing staff: A study based on the theory of planned behavior. *Journal of Management & Organization*, *22*(1), 1-18.	No measure of PS
43. Leroy, H., Dierynck, B., Anseel, F., Simons, T., Halbesleben, J. R., McCaughey, D., ... & Sels, L. (2012). Behavioral integrity for safety, priority of safety, psychological safety, and patient safety: a team-level study. Journal of Applied Psychology, 97(6), 1273.	Objectives measure of PS
44. Marino, M., & Solberg, L. I. (2021). Cultural and structural features of zero-burnout primary care practices: study examines features of primary care	No measure of PS

practices where physician burnout was reported to be zero. *Health Affairs*, *40*, 928-36.	
45. Marshall, T. L., Ipsaro, A. J., Le, M., Sump, C., Darrell, H., Mapes, K. G., ... & Brady, P. W. (2021). Increasing physician reporting of diagnostic learning opportunities. *Pediatrics*, *147*(1).	No Patient Safety outcome
46. Maxfield, D. G., Lyndon, A., Kennedy, H. P., O'Keeffe, D. F., & Zlatnik, M. G. (2013). Confronting safety gaps across labor and delivery teams. *American journal of obstetrics and gynecology*, *209*(5), 402-408.	No measure of PS
47. McCain, N., Ferguson, T., Hultquist, T. B., Wahl, C., & Struwe, L. (2023). Influencing a Culture of Quality and Safety Through Huddles. *Journal of Nursing Care Quality*, *38*(1), 26-32.	does not report on relationship
48. McLinton, S. S., Dollard, M. F., & Tuckey, M. R. (2018). New perspectives on psychosocial safety climate in healthcare: A mixed methods approach. *Safety Science*, *109*, 236-245.	More of qual study than quant
49. Mengstie, M. M., Biks, G. A., Cherlin, E. J., & Curry, L. A. (2023). Organizational culture and barriers to change in University of Gondar Comprehensive Specialized Hospital Cardiac Unit. *BMC health services research*, *23*(1), 296.	No measure of PS
50. Merandi, J., Liao, N., Lewe, D., Morvay, S., Stewart, B., Catt, C., & Scott, S. D. (2017). Deployment of a second victim peer support program: a replication study. *Pediatric quality & safety*, *2*(4), e031.	Not research but program
51. Moake, T. R., Oh, N., & Steele, C. R. (2019). The importance of team psychological safety climate for enhancing younger team members' innovation-related behaviors in South Korea. *International Journal of Cross Cultural Management*, *19*(3), 353-368.	No measure of PS
52. Moraes, M. C. S. D., Dutra, G. O., Ferreira, T. D. M., Dias, F. C. P., Balsanelli, A. P., & Gasparino, R. C. (2021). Nursing coaching leadership and its influence on job satisfaction and patient safety. *Revista da Escola de Enfermagem da USP*, *55*, e03779.	Not in English
53. Moriano, J. A., Molero, F., Laguía, A., Mikulincer, M., & Shaver, P. R. (2021). Security providing leadership: A job resource to prevent employees' burnout. *International journal of environmental research and public health*, *18*(23), 12551.	No measure of PS
54. Niederhauser, A., & Schwappach, D. L. (2022). Speaking up or remaining silent about patient safety concerns in rehabilitation: A cross-sectional survey	Self-report PS

to assess staff experiences and perceptions. *Health science reports*, *5*(3), e631.	
55. Nijs, K., Seys, D., Coppens, S., Van De Velde, M., & Vanhaecht, K. (2021). Second victim support structures in anaesthesia: a cross-sectional survey in Belgian anaesthesiologists. *International Journal for Quality in Health Care*, *33*(2), mzab058.	self-report of QoC
56. Nixon, A. E., Lanz, J. J., Manapragada, A., Bruk-Lee, V., Schantz, A., & Rodriguez, J. F. (2015). Nurse safety: How is safety climate related to affect and attitude?. *Work & Stress*, *29*(4), 401-419.	Self-report*
57. O'Donovan, R., & McAuliffe, E. (2020). Exploring psychological safety in healthcare teams to inform the development of interventions: combining observational, survey and interview data. *BMC health services research*, *20*, 1-16.	No Patient Safety outcome
58. Pfeifer, L. (2022). *Measuring psychological safety, high-reliability (HRO) perception and safety reporting intentions among pediatric nurses* (Doctoral dissertation, Boston College).	Self-report*
59. Pfeifer, L., Vessey, J., Cazzell, M., Ponte, P. R., & Geyer, D. (2023). Relationships among psychological safety, the principles of high reliability, and safety reporting intentions in pediatric nursing. *Journal of Pediatric Nursing*, *73*, 130-136.	Self-report*
60. Prasad, A., Cios, T. J., Staub-Juergens, W., Dziedzina, C., Rao, S., & Singbartl, K. (2020). Standardization improves postoperative patient handoff experience for junior clinicians. *Am J Manag Care*, *26*(6), e184-90.	Self-report*
61. Probst, T. M., & Estrada, A. X. (2010). Accident under-reporting among employees: Testing the moderating influence of psychological safety climate and supervisor enforcement of safety practices. *Accident analysis & prevention*, *42*(5), 1438-1444.	mixed population
62. Raman, R., & Green, K. (2017). Multi-level factors affecting timely electronic documentation of medication administration: a hierarchical linear modeling approach. Health Systems, 6, 171-185.	Objective measure of patient safety
63. Rashkovits, S. (2021). A Likert-Type scale for evaluating the "bottom line" of patient safety. *Journal of Patient Safety and Risk Management*, *26*(1), 29-33.	Self-report*
64. Rathert, C., Ishqaidef, G., & May, D. R. (2009). Improving work environments in health care: test of a theoretical framework. *Health care management review*, *34*(4), 334-343.	Self-report*

65. Reader, T. W., Flin, R., Mearns, K., & Cuthbertson, B. H. (2007). Interdisciplinary communication in the intensive care unit. *British journal of anaesthesia*, *98*(3), 347-352.	No Patient Safety outcome
66. Richard, A., Pfeiffer, Y., & Schwappach, D. D. (2021). Development and psychometric evaluation of the speaking up about patient safety questionnaire. *Journal of patient safety*, *17*(7), e599-e606.	Self-report PS
67. Ridley, C. H., Al-Hammadi, N., Maniar, H. S., Abdallah, A. B., Steinberg, A., Bollini, M. L., ... & Avidan, M. S. (2021). Building a collaborative culture: focus on psychological safety and error reporting. The Annals of Thoracic Surgery, 111(2), 683-689.	Asked if medical error had occurred
68. Schwappach, D. L. B., & Gehring, K. (2015). Frequency of and predictors for withholding patient safety concerns among oncology staff: a survey study. *European journal of cancer care*, *24*(3), 395-403.	Self-report
69. Schwappach, D. L., & Niederhauser, A. (2019). Speaking up about patient safety in psychiatric hospitals–a cross-sectional survey study among healthcare staff. *International journal of mental health nursing*, *28*(6), 1363-1373.	self report
70. Schwappach, D., & Richard, A. (2018). Speak up-related climate and its association with healthcare workers' speaking up and withholding voice behaviours: a cross-sectional survey in Switzerland. *BMJ quality & safety*, *27*(10), 827-835.	Self-report
71. Schwappach, D., Sendlhofer, G., Häsler, L., Gombotz, V., Leitgeb, K., Hoffmann, M., ... & Brunner, G. (2018). Speaking up behaviors and safety climate in an Austrian university hospital. *International journal for quality in health care*, *30*(9), 701-707.	No Patient Safety outcome
72. Schwappach, D., Sendlhofer, G., Kamolz, L. P., Köle, W., & Brunner, G. (2019). Speaking up culture of medical students within an academic teaching hospital: need of faculty working in patient safety. *PLoS One*, *14*(9), e0222461.	No Patient Safety outcome
73. Seibert, M., Hillen, H. A., Pfaff, H., & Kuntz, L. (2020). Exploring leading nurses' work values and their association with team safety climate: results from a questionnaire survey in neonatal intensive care units. *Journal of nursing management*, *28*(1), 112-119.	No measure of PS

74. Sens, F., Viprey, M., Piriou, V., Peix, J. L., Herquelot, E., Occelli, P., ... & IDILIC Study Group. (2022). Safety attitude of operating room personnel associated with accurate completion of a surgical checklist: a cross-sectional observational study. *Journal of Patient Safety*, *18*(5), 449-456.	No Patient Safety outcome
75. Sexton, J. B., Adair, K. C., Leonard, M. W., Frankel, T. C., Proulx, J., Watson, S. R., ... & Frankel, A. S. (2018). Providing feedback following Leadership WalkRounds is associated with better patient safety culture, higher employee engagement and lower burnout. *BMJ quality & safety*, *27*(4), 261-270.	Self report
76. Sholomovich, L., & Magnezi, R. (2017). Tell me how pleased you are with your workplace, and I will tell you how often you wash your hands. *American Journal of Infection Control*, *45*(6), 677-681.	Self report of PS
77. Sinnott, J. (2022). Safety culture, error reporting and medical innovation: comparing a survey taken during COVID-19 to the 2019 national staff survey. *British Journal of Healthcare Management*, *28*(2), 1-7.	No Patient Safety outcome
78. Spânu, F., Băban, A., Bria, M., Lucăcel, R., Florian, I. Ş., & Rus, L. (2013). Error communication and analysis in hospitals: The role of leadership and interpersonal climate. *Procedia-Social and Behavioral Sciences*, *84*, 949-953.	Self report
79. Stühlinger, M., Schmutz, J. B., & Grote, G. (2019). I hear you, but do I understand? The relationship of a shared professional language with quality of care and job satisfaction. *Frontiers in Psychology*, *10*, 423293.	Self-report*
80. Sun, Y., Yang, H., Wu, X., Jiang, Y., & Qian, C. (2022). How safety climate impacts safety voice—Investigating the mediating role of psychological safety from a social cognitive perspective. *International journal of environmental research and public health*, *19*(19), 11867.	Self-report*
81. Tucker, A. L., Nembhard, I. M., & Edmondson, A. C. (2007). Implementing new practices: An empirical study of organizational learning in hospital intensive care units. *Management science*, *53*(6), 894-907.	Self-report*
82. Walther, F., Schick, C., Schwappach, D., Kornilov, E., Orbach-Zinger, S., Katz, D., & Heesen, M. (2022). The Impact of a 22-Month Multistep Implementation Program on Speaking-Up Behavior in an Academic Anesthesia Department. *Journal of patient safety*, *18*(7), e1036-e1040.	Self-report*

83. Wang, W., Zhang, J., Nicholas, S., Yang, H., & Maitland, E. (2023). Organisation-level and individual-level predictors of nurse-reported quality of care in primary care: A multilevel study in China. *Tropical Medicine & International Health*, *28*(4), 308-314.	Self-report*
84. White, S., Barnes, T., Drevo, S., Brower, C., McVey, K. M., & Brancu, M. (2023). Measuring executive leadership teaming dynamics for leading learning organizations. *Consulting Psychology Journal*.	No Patient Safety outcome
85. Wilkens, R., & London, M. (2006). Relationships between climate, process, and performance in continuous quality improvement groups. *Journal of Vocational Behavior*, *69*(3), 510-523.	No Patient Safety outcome
86. Xu, X., Le, N., He, Y., & Yao, X. (2020). Team conscientiousness, team safety climate, and individual safety performance: A cross-level mediation model. *Journal of Business and Psychology*, *35*, 503-517.	Self-report*
87. Yousaf, M., Khan, M. M., & Paracha, A. T. (2022, November). Effects of Inclusive Leadership on Quality of Care: The Mediating Role of Psychological Safety Climate and Perceived Workgroup Inclusion. In *Healthcare* (Vol. 10, No. 11, p. 2258). MDPI.	Self-report*
88. Zhang, R., Gong, Y., & Zhou, M. (2023). Crossing the domain: Unintended consequences of safety and service climates. *Journal of Applied Psychology*.	Self-report*
89. Zhou, P., Bai, F., Tang, H. Q., Bai, J., Li, M. Q., & Xue, D. (2018). Patient safety climate in general public hospitals in China: differences associated with department and job type based on a cross-sectional survey. *BMJ open*, *8*(4), e015604.	No Patient Safety outcome
