# Supplementary material for: Psychological safety and patient safety: A systematic and narrative review
Source: PLoS One. 2025 Apr 24;20(4):e0322215. doi: 10.1371/journal.pone.0322215 (PMC12021220; doi:10.1371/journal.pone.0322215)
Supplement: S5 File — (PDF) [file pone.0322215.s005.pdf]

**Authors (Alphabetical)**	**Country**	**Participants**	**(Total N, % Women)**	**Design**	**Measure of Psychological Safety**	**Patient Safety Outcome**	***Coefficient & Sig.***	**Study Quality[b]**
Anderson et al. (2021)	United States	Mental health (n = 797)<br>Registered Nurses (n = 4331)<br>Licensed Practical Nurses (n = 1518)	6646, NR	Cross-sectional<br><br>Retrospective Database Analysis	Workgroup psychological safety from the "All Employee Survey" within the Veterans Health Administration	The use of seclusion and physical restraining in inpatient psychiatric units.	- *Seclusion Use*<br>β = 2.12, ***p* = 0.03**, 95% CI: [1.08, 4.21] (higher PS → higher seclusion use)<br><br>- *Physical Restraint Use*<br>β = 0.40, ***p* < 0.01**, 95% CI: [0.20, 0.78] (higher PS → lower restraint use)	9/14
Arnetz et al. (2019)	United States	Nurses in the hospital	432/95.1%<br><br>83 blood samples; 95.1%	Cross-sectional<br><br>Retrospective Analysis	Edmondson (1999)	(1) pressure ulcers<br>(2) patient falls<br>(3) central line-associated blood stream infections (CLABSI)<br>(4) catheter-associated urinary tract infections (CAUTI)<br>(5) ventilator-associated events (VAE)<br>- All unit level	Psychological Safety and Stress Biomarkers<br>Individual Nurse Level:<br>- *Serum DHEA-S*<br>*r = 0.22, **p < 0.05***<br>(higher PS → higher levels of the neuroprotective hormone DHEA-S, linked to stress resilience, at Nurse level)<br>-*Serum IL-6*<br>*r* = –0.15, *p > 0.05*<br><br>Psychological Safety and Stress Biomarkers<br>Unit Level:<br>- *Serum DHEA-S*<br>*r = -0.07, p > 0.05*<br>-*Serum IL-6*<br>*r = -0.05, p > 0.05*<br><br>Psychological Safety and Patient Outcomes<br>- *Unit-acquired pressure ulcers*	7/14

							$r = 0.02, p > 0.05$<br>- *Total falls per 1000 patient days*<br>$r = 0.44, p > 0.05$<br>- *CLABSI per 1000 line days*<br>$r = -0.30, p > 0.05$<br>- *CAUTI per 1000 line days*<br>$r = 0.13, p > 0.05$<br>- *VAE per 1000 ventilator days*<br>$r = –0.40, p > 0.05$<br>(PS does not directly correlate with patient safety outcomes)	
Brimhall et al. (2023)	United States	All employees of a non-profit hospital from various departments	N=318 employees from 47 workgroups; NR	Cross-sectional	Edmondson (1999)	Reported medical errors	Psychological Safety and Reported medical errors<br>β = -2.30, $\mathbf{p < .001}$, 95% CI [−3.13, −1.47]	8/14
Gilmartin et al. (2018)	United States	Nurses working in a Veterans Health Administration hospital	2008 1,962; 78.19%<br>2009 1,926; 77.21%<br><br>2010 2,428; 76.89%<br>2011 1,973; 75.98%	Cohort Study - Retrospective Database Analysis	One item from "All Employee Survey"	Nonadherence rates to the central line checklist:<br>(1) hand hygiene before central line insertion<br>(2) application of chlorhexidine gluconate (prep)<br>(3) use of a cap<br>(4) mask,<br>(5) sterile gloves,<br>(6) sterile gown by the provider inserting the central line<br>(7) full-body drape to cover the patient	Psychological Safety and Checklist Compliance:<br>*Nonadherence Rates*<br>p = 0.41<br>*High vs. Low Adherence Units*<br>$p > 0.05$	7/14
Halbesleben et al. (2013)	United States	Registered Nurses	658, 87%	Cross-lagged study	Edmondson's (1999) adapted version (Nembhard	Occupational Injuries	Leader behavioral integrity significantly influences safety compliance and psychological safety, which in turn	9/14

					& Edmondson, 2006)		mediate the effect on safety outcomes (injury frequency, severity, and reporting)<br><br>CFI = 0.97, RMSEA = 0.037<br><br>Cross-Lagged Relationship Findings:<br>Time 1 Psychological safety → Time 2 Reporting Ratio: $\beta = 0.59$, $p < 0.05$<br>Time 2 Psychological safety → Time 3 Reporting Ratio: $\beta = 0.56$, $p < 0.05$	
Jung et al. (2021)	United States	Staff of a Radiation Oncology Department	78, NR	Cross-sectional	Learning Organization Survey (LOS)	Willingness to report incidents (near misses and therapeutic incidents)	Psychological Safety and Near-Miss Reporting:<br>*-Standard Care Scenario*<br>OR: 1.46, 95% CI: 0.78–2.71, *p > 0.05*<br>*-Could Have Happened Scenario*<br>OR: 1.30, 95% CI: 0.91–1.88, *p > 0.05*<br>*-Fortuitous Catch (Near Miss) Scenario*<br>OR: 1.60, 95% CI: 1.10–2.33, ***p* = 0.015**<br>*-Almost Happened (Near Miss) Scenario*<br>OR: 1.60, 95% CI: [1.07–2.37], **p = 0.021**<br>*-Hit (Actual Harm) Scenario*<br>OR: 1.96, 95% CI: [1.19–3.23], ***p* = 0.008**<br><br>(Psychological safety was a stronger predictor of reporting near-misses with greater proximity to harm)	9/14
Leroy et al. (2012)	Belgium	Nurses and Head nurses from various specialty departments	nurses = 580; 75% head nurses = 54; 56%	Cross-lagged	Safety For Nurses: Simons et al. (2007).<br>PS For Teams: Edmondson (1999)	Reported Treatment Errors that resulted in harm to a patient.	Psychological Safety<br>*-Reported Errors*<br>$\beta = 0.28$, ***p* = 0.02**<br><br>Psychological Safety<br>*Priority of Safety → Reported Errors*<br>*β = -0.35*, ***p* = 0.01**	9/14

Raman & Green, (2017)	United States	Non-physician healthcare professionals	803, NR	Cross-sectional	Edmondson (1999)	Medication administration processes in healthcare settings / records	Psychological Safety<br>Individual level<br>- *Timely Medication administration*<br>$\gamma 10 = 0.065$, $p > 0.05$<br>(individual psychological safety did not significantly predict timely medication documentation)<br><br>Psychological Safety<br>Unit level<br>- *Timely Medication administration*<br>$\gamma 04 = 1.309$, $\mathbf{p < 0.001}$<br><br>(unit-level psychological safety a strong and statistically significant predictor)	9/14
Ridley et al. (2020)	United States	Operating Rooms (ORs) Clinicians	73 at Baseline<br>6-month Follow-Up: 68<br>12-month Follow-Up: 68<br>NR	Cohort Study	Edmondson (1999)	Medical Errors Reported During Surgical Cases (defined as a preventable adverse event resulted OR NOT in harm to a patient)	Positive Psychological safety levels:<br>*baseline, 6-month, and 12-month follow-ups*<br>Baseline: 78.1% (57 out of 73)<br>12-Month Follow-up: 88.2% (60 out of 68)<br><br>Psychological safety increased by 10.1%, 95% CI: 2.4%–23.4%, but $p = 0.122$, indicating a non-significant change<br><br>Medical Error Reduction:<br>*baseline, 6-month, and 12-month follow-ups*<br>First 6 Months: 7.44% (78 out of 1048 cases).	11/14

							Second 6 Months: 4.65% (55 out of 1184 cases).<br>Error rates decreased by 2.79%, 95% CI: 0.8%–4.8%, ***p* = .005**, indicating a significant change	
