# Supplementary material for: Psychological safety and patient safety: A systematic and narrative review
Source: PLoS One. 2025 Apr 24;20(4):e0322215. doi: 10.1371/journal.pone.0322215 (PMC12021220; doi:10.1371/journal.pone.0322215)
Supplement: Attachment — Submitted filename: Response to reviewers.docx [file pone.0322215.s008.docx]

Response to reviewers

Dear Dr. Connolly and Reviewers,

We want to thank you and the three reviewers for their constructive feedback on our paper. Their suggestions improved the paper. In the following letter, we address all three reviewers' comments in detail and provide a revised manuscript using ‘track changes’ to indicate where the paper has changed.

**Reviewer #1**

1. Major: The problem analysis does not fit the research question. Bade on the introduction, the reader expects the paper to focus on the complex relationship between PS and outcomes and especially the underlying mechanisms. Instead the paper focuses on the evidence of the relationship and its implications for interventions.

Our Response: We have rewritten the introduction section to better reflect the complex relationship between PS and various outcomes.

2. Following the introduction, the research question should be wider and especially include the subjective patient safety outcomes in order to unravel this complex relationship. OR the introduction should be complete rewritten in order to focus on why we need a summary of evidence for this specific relationship, why is it so important to show the reader this overview of evidence. In addition, also argue why this overview does not include the subjective safety outcomes.

Our response: Previous systematic reviews on psychological safety in healthcare (i.e., Grailey et al, 2021; O’Donovan & McAuliffe, 2020) have reviewed papers that reported on the relationship between psychological safety and self-reported outcomes. However, it is widely acknowledged that self-report survey measures are limited by self-report bias and response fatigue (Donaldson & Grant-Vallone, 2002: Newman et al, 2017). Moreover, there is evidence that self-reports can artificially inflate the relationship between safety climate and safety outcomes (Beus et al., 2010), whereas evaluations of hospital patient safety performance (as well as fines, suspensions and closures) are based on objectively reported metrics (e.g., medical errors, critical incidents). Our aim in this paper was to provide a new contribution to the field in line with the current literature highlighting the complexity of defining patient safety and the need for updated approaches to patient safety definitions. Thus, we focused on studies that reported the relationship between PS and objective/robust measures of patient safety. Recent reviews of the PS area report on the positive significant relationships between high levels of psychological safety with learning and creativity outcomes (e.g., Edmondson & Bransby, 2023). However, this trend is not observed in healthcare (we cite the relevant research in our introduction). Therefore, we believe that a review of the evidence on the relationship between psychological safety and objective measures of patient safety would provide a new and needed addition to the literature. We hope that the reviewer agrees with our sentiments. We would also like to add that our collective experiences of teaching healthcare professionals indicate a desire from healthcare workers to be informed of the evidence concerning the impact of psychological safety on patient safety ‘in practice’.

References

Beus JM, Payne SC, Bergman ME, Arthur Jr W. Safety climate and injuries: an examination of theoretical and empirical relationships. Journal of applied psychology. 2010 Jul;95(4):713.

Donaldson SI, Grant-Vallone EJ. Understanding self-report bias in organizational behavior research. J Bus Psychol 2002;17:245–60.

Edmondson AC, Bransby DP. Psychological safety comes of age: observed themes in an established literature. Annual Review of Organizational Psychology and Organizational Behavior. 2023 Feb 2;10(1).

Grailey KE, Murray E, Reader T, Brett SJ. The presence and potential impact of psychological safety in the healthcare setting: an evidence synthesis. BMC health services research. 2021 Dec;21:1-5.

Newman A, Donohue R, Eva N. Psychological safety: a systematic review of the literature. Hum Resour Manag Rev 2017;27:521–35.

O’donovan R, Mcauliffe E. A systematic review of factors that enable psychological safety in healthcare teams. International journal for quality in health care. 2020 May;32(4):240-50.

3. Minor: Why is there a heading called “psychological safety and psychological safety”

Our response: Thank you for pointing this out. Apologies, we have corrected this typo.

4. Methods: In the future you might consider using tools to assist you in a systematic review, such as Ryyan or ASreview

Our response: We agree with the point of the reviewer. However, please note that we did use Rayyan in our review process. See - “Eligibility Criteria and Study Selection,” “Duplicate control and title and abstract review were conducted using Rayyan”.

5. You might consider identifying other reviews on PS. What did and didn't they conclude. Did they also include your selection of papers. Have you identified new papers or was this review also be able to be conducted based on the present reviews?

Our response: We have cited and included a large number of reviews on PS in our paper (see list below). The papers were useful in identifying the gaps in the literature. We highlight in the introduction the added value of our paper – in that it addresses an area that has not been previously covered in the literature. As we have previously mentioned in point 2, review papers to date have highlighted the problem surrounding the overreliance on self-report measures in terms of common method variance.

Here is a list of the included review papers in our manuscript:

Edmondson AC, Lei Z. Psychological safety: The history, renaissance, and future of an interpersonal construct. Annual Review of Organizational Psychology and Organizational Behavior. 2014 Mar 21;1(1):23–43.

Edmondson AC, Bransby DP. Psychological safety comes of age: observed themes in an established literature. Annual Review of Organizational Psychology and Organizational Behavior. 2023 Feb 2;10(1).

Frazier ML, Fainshmidt S, Klinger RL, Pezeshkan A, Vracheva V. Psychological safety: A meta-analytic review and extension. Personnel Psychology. 2017 Oct 14;70(1):113–65.

Grailey KE, Murray E, Reader T, Brett SJ. The presence and potential impact of psychological safety in the healthcare setting: an evidence synthesis. BMC health services research [Internet]. 2021 Aug 5 [cited 2021 Nov 4];21(1):773.

Liu JW, Ein N, Plouffe RA, Gervasio J, St. Cyr K, Nazarov A, Richardson JD. Meta-Analysis and Systematic Review of the Measures of Psychological Safety. medRxiv. 2024:2024-02.

O’donovan R, Mcauliffe E. A systematic review of factors that enable psychological safety in healthcare teams. International Journal for Quality in Health Care. 2020 Mar 31;32(4):240–50.

6. Results: - In the interpretation of the relationship there is a lack of nuanced. If a relationship between PS and medical errors is found, this means that people are more or less willing to report errors, it is not the same as the actual errors made. One could argue both ways: high PS could make the reporting of errors less urging and therefore lower OR high PS could make the reporting of errors more natural in an open and learning environment. This is somewhat later on discussed but not incorporated in the presentation of the results at the beginning of the Result section.

Our response: We thank the reviewer for the opportunity to improve the quality of the Results section. We have now addressed the difficulties of a “straightforward interpretation” at the beginning of the Result section entitled - (“Is there a relationship between Psychological Safety and Patient Safety?) section”.

We have included the following text: “PS and positive objective patient safety outcomes are sometimes “difficult” to detect and resistant to linear interpretation, as PS seems to share contextual dependencies between objective reporting behavior and patient safety. Additionally, high reporting rates may indicate high PS, where incidents are openly discussed and used as learning opportunities and protocol compliance. Conversely, low reporting rates might be interpreted as evidence of enhanced team performance, although this could also reflect underreporting or fear of disclosure.”

7. There is a lack of insights presented in teamwork in healthcare. People that filled in the questionnaire of Edmondons are healthcare professionals. They are part of multiple teams; multi teammembership. What are the teams we are referring to in the result section. Are that the multidisciplinairy teams that provide care? Or the monodisciplinairy teams? That is a big difference in relation to PS. In multidisciplinairy teams the different disciplines are stables but not the people representing this discipline.

Our response: We agree with the point of the reviewer. We do mention this as one of the limitations of the Edmondson questionnaire (see our section on Methodology and Sampling Issues), in that it's not possible to know what ‘team’ respondents have in mind when they fill in the questionnaire. The teamwork element is an important factor in this story, but our review of the nine papers did not allow us to reach any substantive conclusions on this issue.

8. Discussion/conclusion: What is the answer to the second part of the research question? Not clear what this review adds. The paradoxes where already know prior to the research. Make clear what we did not know and after the review do know. After rewriting the introduction, have in mind that the introduction and discussion should cover other angles. Now they are too similar in their message.

Our response: We hope that our rewriting of the paper has illuminated more clearly what our review has contributed to the field. We agree with the reviewer that the paradoxes were already mentioned in the literature, however – we would argue that our paper is the first attempt to review the paradox between PS and patient safety in detail. Beyond this, we can provide a clearer description of what our paper contributes:

**What is already known on this topic** – High levels of psychological safety in healthcare teams has been advocated as an important mechanism by which patient safety can be improved. However, the majority of the research is based on self-report measures.

**What this study adds** – This is the first systematic review to assess the relationship between psychological safety and objectively measured patient safety. It identifies that commonly used definitions of patient safety as simply the absence of harm, might need to broaden to concepts of contemporary safety science.

**How this study might affect research, practice or policy** – High levels of psychological safety has been advocated as an important mechanism by which patient safety can be improved, but there is insufficient evidence to support this idea due to the framing of safety only as the absence of harm in the healthcare literature, rather than a safety science lens

**Reviewer #2:**

1. This is a really interesting and important paper and congratulations to the authors. The comments made here are in the spirit of strengthening the paper.

Our response: We appreciate the reviewer’s perspective, and we thank for the positive appraisal of the revised manuscript, as well as for the opportunity to further enhance the quality of the paper.

2. The definition of psychological safety is important. Edmondson’s 1999 definition and measurement is not just about me being able to take risks (express vulnerability) in relation to what I do and do not know but also relates to me being able to question my colleagues on their actions and how correct/safe they are. E.g. it is an important point made in the introduction that in healthcare a lot of time is spent trying to figure out what is safe to talk about – but is this not what psychological safety is about?

Our response: We agree with the point of the reviewer that the Edmondson approach is about taking risks but also relates to speaking up to colleagues. We acknowledge this problem in terms of our discussion of the inherent challenges in using the Edmondson questionnaire and what it means for the validity of psychological safety. For example, we mention the problem of what individuals have in mind when thinking about what ‘team’ the questionnaire refers to. Additionally, we note the reliability and unidimensional limitations of the Edmondson questionnaire. The point about speaking up is an important one, but the available research evidence in organizational behaviour literature tends to treat employee silence and employee voice as distinct constructs from psychological safety. However, this is a bigger problem concerning silence and voice in healthcare per se, with a recent integrative review indicating a need for further research regarding the distinction between what drives safety voice versus general employee voice, and how both voice and silence can operate in parallel in healthcare (Lainidi et al, 2023). We agree with the reviewer that a lack of psychological safety means either uncertainty on whether doing the right thing will be accepted or even certainty that other issues (e.g., the reputation of the unit/hospital) should be prioritised over doing the right thing by e.g., the patients. In that sense, speaking up can be included under the “taking a risk” umbrella. In practical terms, healthcare professionals might experience the high levels psychological safety in their team/unit, while also knowing there are certain topics that should not be voiced.

Lainidi O, Jendeby MK, Montgomery A, Mouratidis C, Paitaridou K, Cook C, Johnson J, Karakasidou E. An integrative systematic review of employee silence and voice in healthcare: what are we really measuring?. Frontiers in Psychiatry. 2023 May 25;14:1111579.

3. There are more nuanced definitions of patient safety other than freedom from harm and as you argue this is not a good definition. I think these other definitions informed by Safety Management Systems work in aviation and other fields should also be included here?

Our response: We thank the reviewer for this suggestion. We have now extended the definition to state: “Defining the commitment to advancing safe care, patient safety—the most enduring and foundational principle of medicine—represents the core value of healthcare quality by emphasizing freedom from any harm associated with health care in clinical practices.”

4. Section – Psychological safety and should be ‘patient’ safety?

Our response: Thank you for pointing this out. Apologies, we have corrected this typo.

5. Line 80 - Medical errors third leading cause of death – needs to be nuanced. https://qualitysafety.bmj.com/content/26/5/423

Our response: We thank the reviewer for this advice. We have now corrected the percentage to “Medical errors are translating into over three million deaths globally each year” as reported in Global patient safety report 2024. Geneva: World Health Organization; 2024.

6. Lines 85-90 Should psychological safety not also lead to increased reporting of adverse events and to increased challenges to each other on unsafe practices?

Our response: We thank the reviewer for raising this crucial point. In healthcare, where professionals are highly trained and educated, psychological safety *should* lead to increased reporting of adverse events/incidents/errors and more frequent challenges to unsafe practices only when healthcare professionals perceive reporting as a constructive action aimed at improving patient safety and delivering high-quality care, rather than as an admission of personal failure or accountability. However, this association seems to be quite complex as psychological safety could also lead to decreased reporting of adverse events/incidents/errors, while also there is not concrete evidence on “how much” psychological safety is needed or whether too much psychological safety can lead to unnecessary risks or aversion towards formal reporting. We agree with this point and it links to the reviewer’s earlier comment on what psychological safety is – and this point indirectly implies that academics and practitioners might need to think what is NOT psychological safety. To address this, in the Results section (at the beginning of - Is there a relationship between Psychological Safety and Patient Safety?) we have added the following text:

“PS and positive objective patient safety outcomes are sometimes “difficult” to detect and resistant to linear interpretation, as PS seems to share contextual dependencies between objective reporting behavior and patient safety. Additionally, high reporting rates may indicate high PS, where incidents are openly discussed and used as learning opportunities and protocol compliance. Conversely, low reporting rates might be interpreted as evidence of enhanced team performance, although this could also reflect underreporting or fear of disclosure.”

7. Line 112 – why were only quantitative studies included – needs justification

Our response: We thank the reviewer for this suggestion. We have now provided greater clarity “This systematic review included only studies with robust quantitative indicators as they employ systematic indicators and epidemiological methods to quantify specific aspects of objective patient safety measures, including the use of the reporting system and checklists, adverse events, harm to patients and providers, and risks associated with adverse events. ”

8. Results: Line 138 not sure why female participants pulled out in particular – would it be more appropriate to say ‘Only 4 out of 9 studies noted gender of participants’.

Our response: We thank the reviewer for this suggestion. We have now revised the sentence “Gender representation was noted in only 44% (4 out of 9) of the studies”

9. Line 142 needs more discussion on what tools other than Edmondson were used.

Our response: We have included information on the other tools used in the remaining studies.

10. Discussion. Line 279 – or improved objective patient safety measures (rather than just formal reporting or errors?) and what are these?

Our response: We thank the reviewer for this suggestion, we have now added “improved objective patient safety measures” as recommended. The updated text addresses these measures, including “the use of the reporting system and checklists, adverse events, harm to patients and providers, and risks associated with adverse events”

**Reviewer #3:**

1. This an important systematic review of the evidence linking psychological safety and patient safety. The results will be of great interest to specialists in the patient safety domain.

Our response: We appreciate and thank the reviewer for the positive appraisal of the manuscript topic and for providing the opportunity to further enhance the quality of the paper.

2. I think the manuscript could be greatly improved if the authors were to present their review from a different stance. Rather than presenting a list of the findings of the nine studies, I suggest a more nuanced approach which recognises the complexity of the link between the two entities. We know already that healthcare teams with high psych safety can appear to be operating less safely because they tend to report more patient safety incidents and unsafe practices. This could be the main focus of the research rather than a straight forward yes/no research question. The sections towards the end of the manuscript nicely address this and they could be moved to the beginning of the manuscript as the background to the review.

Our response: We thank the reviewer for the suggestion. We have taken a more narrative approach to the paper and significantly rewritten the introduction and discussion sections. As suggested, we have taken a more nuanced approach to the complex link between the two entities. That said, we have been careful not to exaggerate the conclusions of our paper, given that they are based on nine papers.

4. I think the results section should tell a 'story' rather than a 'a laundry list' of study findings. The study findings could be used as evidence for the 'story'. This would address an issue in the content where the authors report the study findings and then make a conclusion. It would be more interesting to reverse this process.

Our response: We thank the reviewer for this insightful suggestion and the opportunity to clarify the results section further. We have now reversed the process. The study findings are now presented as evidence, aligning closely with the “story – review’ objectives”'

5. In my view (a) and (b) in the research question are not necessary and take from the impact of the question itself.

Our response: We thank the reviewer for the opportunity to clarify this point further. We removed (a) and (b) from the research question to enhance the clarity and impact of the question itself. As recommended, we have now rephrased the research question to avoid oversimplification and better reflect the complexity of the outcomes.

6. I also have some typos and writing style issues spotted which I hope are helpful: The 0-10 rule is inconsistently applied. i.e. numbers between 0-10 should be written in long hand (abstract and L139, 8/9 7/9 etc )

Our response: Thank you. We have corrected these issues.

7. L57, Should 'of' be deleted ?

Our response: We have deleted ‘of’ in this sentence.

8. Ideally sentences that begin with XXXXX found/demonstrated ........are not ideal....Instead state the finding and include the author reference at the end where possible.

Our response: We agree with the reviewer. We have only named the authors at the beginning of the sentence when discussing the nine studies included in the review. We think this makes it easier for reader to link this section with Table 1.

9. L 151 inconsistent use of capital letters

Our response: Thank you. We have corrected this.

10. L92 rewrite' Therefore the direction or existence of causality between patient and PS is not clear'.

Our response: Thank you. We have corrected this.

11. Psychological safety is shortened early on to PS but this is not then used consistently ie Lines 96,207,209,214,228,255,256,259,262,264,268,270,273,274,277,282,296,304,307,318,325,330,331,334,337,336,337,338. Personally I prefer the phrase rather than the acronym as its easier for the reader.

Our response: We have consistently used the acronym “PS” throughout the manuscript.

12. L 225 paper or papers?

Our response: Thank you. We have corrected this.

13. I would love to see this manuscript published as it would add value to the body of knowledge if it was to be rewritten more creatively.

Our response: We appreciate the reviewer’s positive appraisal and have revised sections of the manuscript as suggested.
